# Proceedings of Fox River Valley Association

**Published:** 1865-11

**Authors:** D. W. Young, E. Winchester

**Affiliations:** Pres’t.; Sec’y.


### Proceedings of Societies.

### PROCEEDINGS OF F. R. V. ASSOCIATION.

The Fox River Valley Medical Association met in the Common Council Room, in the City of Aurora, on Monday afternoon, Oct. 2d, 1865.

The President, Dr. Tefft, in the chair.

Present Drs. Tefft, Long, LeBarron, Eddy, Cushing, Winchester, Pieronett, Hance, Howell, Hawley, Hard, Winslow, Jenks and Young.

Minutes of last meeting read and approved.

Dr. D. W. Young proposed Drs. Abner Hard, George Oldmixon, and S. R. Millard, as suitable persons to become members of the Association.

On motion of Dr. Winchester, they were ballotted for and elected.

Dr. Hard came in, paid the admission fee, signed the constitution, and became a member of the Association.

Dr. Wm. LeBarron, of Geneva, proposed the following questions for discussion:—

1st. Are there any forms of continued fever, bilious or otherwise, in which there is no alternations of chills with the fever, which can be broken up by quinine? In other words, is the recurrence of chills, not only in the beginning, but also in the course of the fever, essential to make that kind of fever usually considered of miasmatic origin, in which quinine is a specific remedy?

2d. Has quinine intrinsically any febrifuge properties, or does it ever allay or diminish fever except in those cases of intermittent or remittent fever in which it is known to be a specific remedy?

3d. In case of doubt to as to the intermittent or miasmatic nature of a fever, is quinine a harmless medicine to experiment with, or is it liable to produce serious congestion of the brain or lungs, either or both?

A lengthy and interesting discussion ensued, which was participated in by most of the members present. Finally, on motion of Dr. Hawley, of Aurora, these questions were continued for discussion at the next meeting, and Dr. W. LeBarron, of Geneva, appointed to open the discussion by a written address.

Dr. Young, of Aurora, reported a case of diphtheria that he was called to see in consultation, where the disease extended to, and was largely expended upon, the larynx. The formation of membrane had been very rapid, and the expectoration profuse, notwithstanding the patient had been early and energetically treated by a very intelligent and competent practitioner.—The patient died, mostly from suffocation. He desired to know the opinions of the members of the Association, whether the operation of tracheotomy would be of any use in such cases and justifiable.

Dr. Winchester, of Elgin, thought that in such recent and rapid cases the operation would be justifiable, and might be beneficial, notwithstanding all the cases thus far reported as having been operated on have died. Most of the members thought the operation useless, as experience thus far has taught that all who have been operated on have died.

Dr. Young referred them to several recently reported cases where the operation had been performed, and the patient died. He deemed the operation in this disease useless, and therefore unjustifiable.

On motion of Dr. Eddy, the Association proceeded to the election of officers.

The President appointed Drs. Hawley and LeBarron as tellers.

The following named gentlemen were elected as officers for the ensuing year:—

President, D. W. Young, M.D., of Aurora.
Vice-President, N. P. Eddy, M.D., of Geneva.
Secretary, E. Winchester, M.D., of Elgin.
Treasurer, S. O. Long, M.D., of Big Rock.

Executive Committee, Drs. S. B. Hawley, of Aurora; D. Jenks, of Plano; A. Pieronett, of Batavia.

The retiring President, Dr. Tefft, then read his valedictory address, in which he named every regular physician who has lived and practised in Kane County since its organization as a county.

On motion of Dr. Winchester, a vote of thanks was tendered to Dr. Tefft for the able manner in which he had discharged the duties of his office for the past year.

On motion of Dr. Hawley, a vote of thanks was tendered the retiring President for his able and interesting address, and Drs. Winslow, of Aurora, and LeBarron of Geneva, were appointed a special committee to assist Dr. Tefft in securing the further history of the regular physicians of the county, and in securing the publication of the same in the county papers.

On motion of Dr. Young, the Junction, in DuPage County, was selected as the next place of meeting.

Dr. Winchester, of Elgin, suggested that the January meeting coming during the holidays, we, as an Association, ought to get up some social entertainment in the evening.

On motion of Dr. Eddy, the Association adjourned to meet at the Junction, on the second Monday afternoon in January, 1866, at 1 o'clock.

Dr. D. W. YOUNG, Pres't.

Dr. E. Winchester, Sec'y.

